# Luminescent Hydroxyapatite Doped with Rare Earth Elements for Biomedical Applications

**DOI:** 10.3390/nano9020239

**Published:** 2019-02-10

**Authors:** Ionela Andreea Neacsu, Alexandra Elena Stoica, Bogdan Stefan Vasile, Ecaterina Andronescu

**Affiliations:** Faculty of Applied Chemistry and Materials Science, Politehnica University of Bucharest, 1-7 Polizu Street, Bucharest 011061, Romania; neacsu.a.ionela@gmail.com (I.A.N.); oprea.elena19@gmail.com (A.E.S.); ecaterina.andronescu@upb.ro (E.A.)

**Keywords:** luminescent materials, hydroxyapatite, bioimaging, rare earth elements

## Abstract

One new, promising approach in the medical field is represented by hydroxyapatite doped with luminescent materials for biomedical luminescence imaging. The use of hydroxyapatite-based luminescent materials is an interesting area of research because of the attractive characteristics of such materials, which include biodegradability, bioactivity, biocompatibility, osteoconductivity, non-toxicity, and their non-inflammatory nature, as well their accessibility for surface adaptation. It is well known that hydroxyapatite, the predominant inorganic component of bones, serves a substantial role in tissue engineering, drug and gene delivery, and many other biomedical areas. Hydroxyapatite, to the detriment of other host matrices, has attracted substantial attention for its ability to bind to luminescent materials with high efficiency. Its capacity to integrate a large assortment of substitutions for Ca^2+^, PO_4_^3−^, and/or OH^−^ ions is attributed to the versatility of its apatite structure. This paper summarizes the most recently developed fluorescent materials based on hydroxyapatite, which use rare earth elements (REEs) as dopants, such as terbium (Tb^3+^), erbium (Er^3+^), europium (Eu^3+^), lanthanum (La^3+^), or dysprosium (Dy^3+^), that have been developed in the biomedical field.

## 1. Introduction

Bioactive inorganic materials have attracted significant attention for their use in different clinical applications. A good example of the need for such materials is represented by the demand for synthetic biomaterials to restore or/and replace bone tissue lost from damage or disease, which has considerably increased in the last few years [1].

The nanoscale of such luminescent materials has gained a lot of importance due to a growing need for valuable photosensitive materials for modern optoelectronic or photonic tools, above and beyond the applications in just the biomedical field. In the biomedical field, luminescent materials, which, for the most part, include fluorescent molecules (organic especially) and semiconductor nanomaterials, have been extensively explored for uses in biological staining and prognostics [2].

Currently, considerable focus has been concentrated on the lanthanide elements to provide photoluminescent materials in bio-technological and/or electronic areas of research. The lanthanide ions are well known for their photoluminescent properties in the visible and near-infrared regions [3].

Different luminescent nanoparticles and nanomaterials have been utilized in the imaging of tissues and intracellular structures. However, in order to develop increased applications in living cells, it is fundamental to develop new biocompatible nanomaterials that can be viewed under visible light [4].

Photoluminescent probes also represent an important domain in chemical and biological research as a result of their capacity to detect particular species with great sensitivity and specificity. In order to achieve lower detection limits, it is important to find methods of increasing their performance [5]. 

The improvement of probes for biomedical applications requires special materials with specific characteristics (e.g., low toxicity levels) apart from fluorescence or magnetic ability in order to be observed by confocal microscopes or magnetic resonance imaging (MRI) [6].

Calcium phosphate coatings have a superior ability to absorb proteins into biomaterial surfaces, which is beneficial for early healing events at the tissue or material interface, leading to expanded bone formation. Among calcium phosphates, hydroxyapatite coatings represent a promising vehicle for improving osseointegration [7].

Hydroxyapatite (HA; molecular formula: Ca_10_(PO_4_)_6_(OH)_2_) represents the major inorganic constituent of human hard tissues and has been extensively used in bone and teeth reconstruction considering its superior biocompatibility and osteoinductivity [1,8].

**Figure 1:**
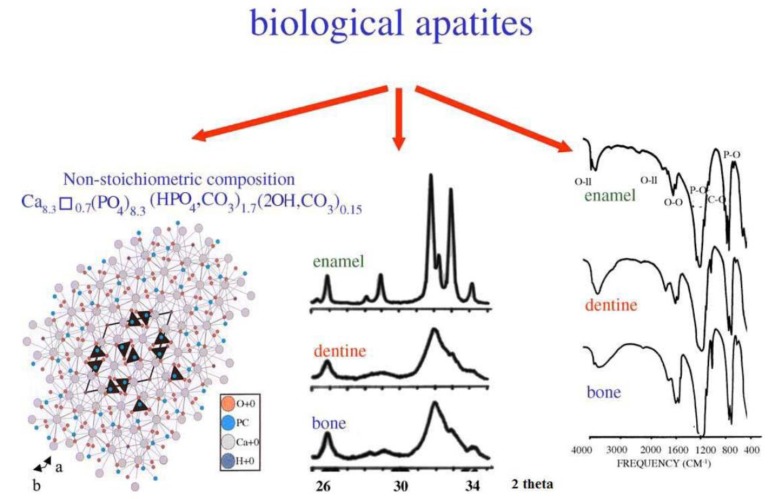
Crystal structure of biological apatites. Powder X-ray diffraction patterns and infrared spectra of enamel, dentine, and bone [11].

Biological apatite is a nonstoichiometric calcium phosphate containing ions, such as Na^+^, Mg^2+^, or CO_3_^2−^ (Figure 1). Biological calcium phosphate is a nanomaterial with a low crystallinity degree. Synthetic hydroxyapatite is a stoichiometric material with a Ca/P ratio of 1.67. Thanks to its excellent osteoconductive characteristics, hydroxyapatite is extensively used as a bioceramic in different clinical applications, such as bioceramic coating, bone tissue engineering, drug and gene delivery, and dental applications [9,10].

The present methods for obtaining hydroxyapatite mostly include conventional template synthesis [12,13], wet chemical synthesis [14,15,16], hydrothermal conversion of calcium carbonate exoskeleton [17,18,19], calcination of xenogenic bone [20,21], solid phase reactions [22], co-precipitation reactions [23,24,25], sol–gel processes [26,27,28], biomimetic preparation [29,30,31], and pyrolysis [31,32,33]. The properties of synthetized hydroxyapatite differ based on preparation route, precursors, etc. [34].

**Figure 2:**
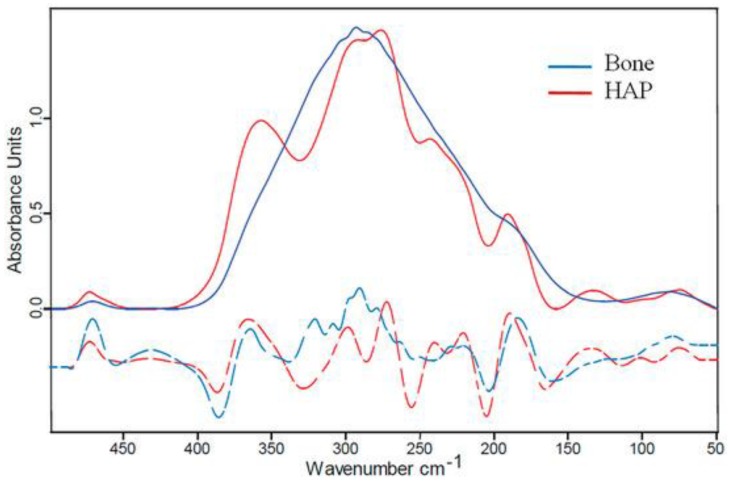
Far-IR (Far InfraRed) spectroscopy of bone. Blue line: bovine bone; red line: hydroxyapatite (HAP) powder [35].

Biological apatite differs considerably from synthetic hydroxyapatite because of its stoichiometry, composition, and crystallinity (Figure 2). The resorption process of synthetic hydroxyapatite is slow due to its greatly crystalline character, and for this reason, it has a low capacity to instantly form a connection with bone tissue or to promote new bone development. Thus, synthetic hydroxyapatite is not appropriate for decreasing the convalescence time of the subject. Furthermore, synthetic hydroxyapatite presents inferior reactivity with the bone environment because of its slower rate of bone integration and apposition [9].

**Figure 3:**
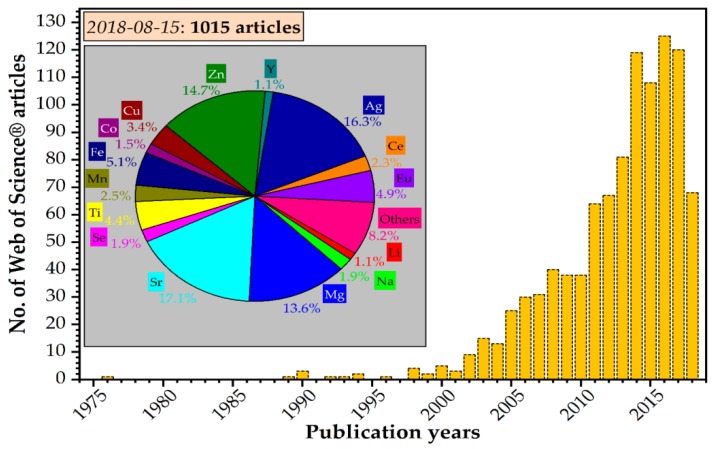
Yearly distribution of scientific articles published on the doped/substituted hydroxyapatite topic in 1975–2018 (as of 15 August 2018) [36].

To reduce all these obstacles, synthetic hydroxyapatite can be adapted with the help of ionic substitution by replacing the Ca^2+^, PO_4_^3−^, or OH^−^ ions. Different ionic substitutions in the hydroxyapatite crystal structure (Ca_10−*x*_M*_x_*(PO_4_)_6_(OH)_2_) have been performed to modify its properties, as can be observed in Figure 3 [9]. 

These substituted ions have certain impacts on the character of the structure of hydroxyapatite, affecting the degree of crystallinity, crystallite size, solubility, thermal stability, lattice parameters, morphology, and bioactivity of hydroxyapatite. It is well known that substituting a modest quantity of Ca^2+^ ions in the hydroxyapatite structure could contribute to lattice disorder, diminish the particle size, reduce the crystallinity degree, and promote a bioresorption process. The existence of certain dopants in hydroxyapatite structure can also lead to an accelerating discharge of Ca^2+^ ions in the enclosing fluid, which can promote fast deposition of apatite on the surface of the pattern [9].

In the research of biological processes in living cells, it is essential to examine any modification in situ in real time. Biological probes are used as one of the most efficient instruments for biological staining and diagnostics. Hence, the progress of developing innovative biological probes currently receives extensive attention [37].

The field of nanotechnology has been considered to be the fastest rising trend in the future of medical care. Currently, lanthanide-doped nanoparticles are being studied as a new category of luminescent optical labels that could represent a promising alternative to organic fluorophores and quantum dots for use in the biomedical field, especially considering their great quantum yield, considerable Stokes shift, enlarged lifetime, and great stability. Nevertheless, there are some important research challenges that have yet to be solved, such as obtaining a perfect biocompatible and biodegradable imaging probe based on nanoparticles for medical use [38].

Biocompatible luminescent materials represent a suitable implant material because of their great potential in biomedical applications. For instance, luminescent marking embodies a promising approach for achieving nondestructive in vivo observation. Moreover, luminescent properties could be induced into hydroxyapatite by replacing the calcium (Ca^2+^) ions with different luminescent rare-earth elements (Table 1) [9].

## 2. Hydroxyapatite Doped with Photoluminescent Elements

Photoluminescence is a very important and useful mechanism in in situ investigations for tissue engineering, surgery, tissue restoration, etc. Labeling with the aid of organic fluorescent molecules has been popular in clinical trial for years. In recent times, many inorganic components, even nanoparticles, have been proposed to be such candidates. Nonetheless, the toxicity of such particles represents a challenge to practical application because of their composition and nano-size character. A luminescent material with high biocompatibility is a perfect candidate for implantation and clinical application. Not only is biocompatibility an important issue, a longer luminescent lifetime is a significant benefit in practical applications as well [39].

Photo-induced luminescent phenomena are represented by fluorescence (decay time <10 ms) and phosphorescence (decay time >0.1 s). According to their composition, photoluminescent (PL) materials can be split into organic and inorganic categories. Inorganic materials from elementary processes usually have longer lifetime and better stability. Generally, for organic materials under sustaining metabolism, the effective fluorescent time ranges from minutes to hours, which limits the use of such photoluminescent compounds in diagnosis applications [39].

Unfortunately, pure hydroxyapatite is not able to exhibit substantial fluorescence under visible excitation. Furthermore, it is widely accepted that Ca^2+^ ions do not possess notable luminescent ability. However, due to their great biodegradable characteristics, hydroxyapatite nanoparticles may serve as an excellent candidate for biological imaging and drug delivery. Prior research has proposed that calcium phosphate nanoparticles could be utilized as fluorescing probes after doping with rare earth elements [4]. Hence, it is also indicated that hydroxyapatite could exhibit fluorescence under visible lights if it is doped with fluorescent ions, of which Eu^3+^ and Tb^3+^ ions are the most intense emitting elements. Nonetheless, the particle sizes and/or the fluorescent radiation in the cells cannot be very well supervised, which limits the function of these materials [37]. 

**Figure 4:**
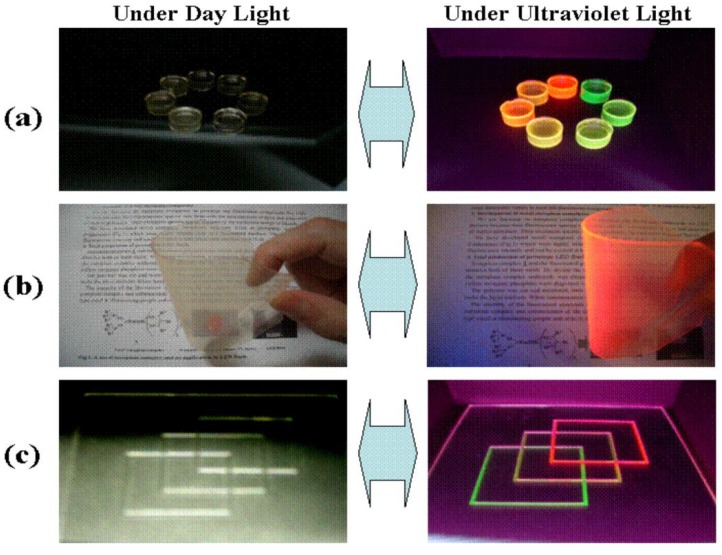
Colorless and transparent emission materials produced by dissolving Eu(III) complexes and/or Tb(III) complexes in a polymer. (**a**) block; (**b**) flexible sheet; (**c**) print on glass [43].

The luminescent character of rare earth elements has been extensively applied, because they can work as visible and near-IR radiation sources, mainly when they dope phosphate compounds such as apatite [40]. Rare earth elements (REEs) is the term frequently used for the elements from lanthanum to lutetium (Z numbers 57–71). REEs have attracted increasing attention in the past decade as a result of their wide applications (e.g., magnets, catalysts, electronics, alloys, ceramics, etc.) [41]. Nowadays, rare earth elements are of significant importance for the global economy. In particular, REEs are important in new technologies, such as LCD (liquid-crystal display), batteries, catalytic converters, and green technologies (e.g., wind turbines) (Figure 4) [42].

**Figure 5:**
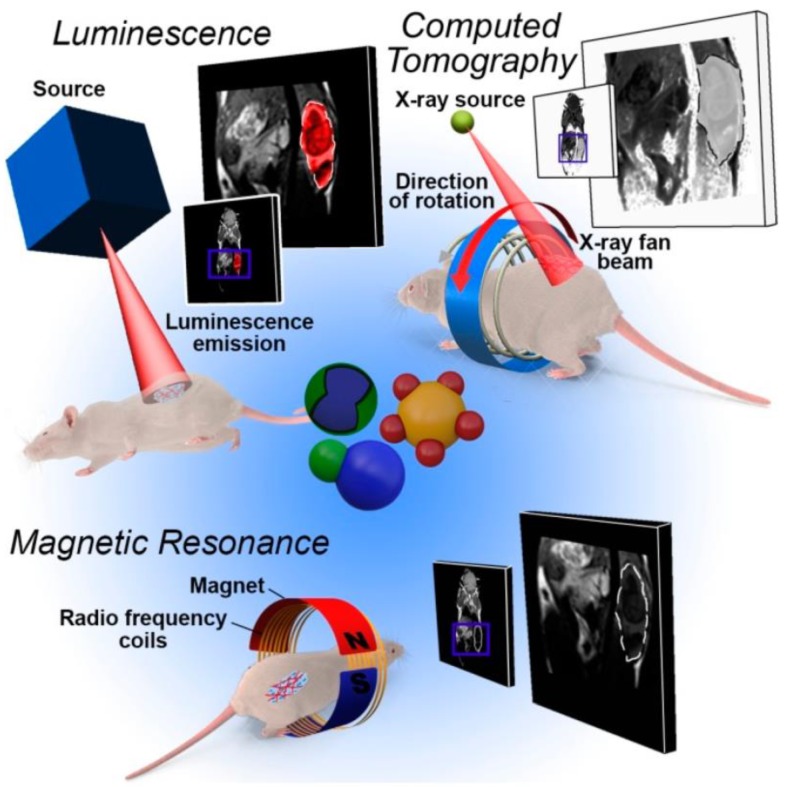
Overview of the imaging techniques most used in heterostructure-based diagnostics. Owing to the multiple domain structure, the patient model can be examined by different imaging methods after the administration and delivery of a single nanostructure [46].

Luminescent rare earth probes present a highly surprising spectral nature that makes them favorable as a nonisotopic substitute for organic fluorophores, especially where there are complications from background autofluorescence. The applications of this class of materials vary from in vivo detection of cellular function and luminescent labeling of biologically related molecules to the clarification of the structure and function of proteins and enzymes (Figure 5). Even if the luminescent properties of aqueous solutions of elementary salts of rare earth ions (e.g., Tb^3+^, Eu^3+^) are relatively poor because of their small absorption cross-section in the UV–visible domain, the particular emission characteristics of these metal ion (Tb^3+^, Eu^3+^) complexes have been applied in biological systems [44]. It is well known that rare earth elements concentrate in marine phosphorite deposits during their formation process, mainly through early diagenetic processes where carbonates are substituted by carbonate fluorapatite (CFA), which also precipitates in void space [45].

**Figure 6:**
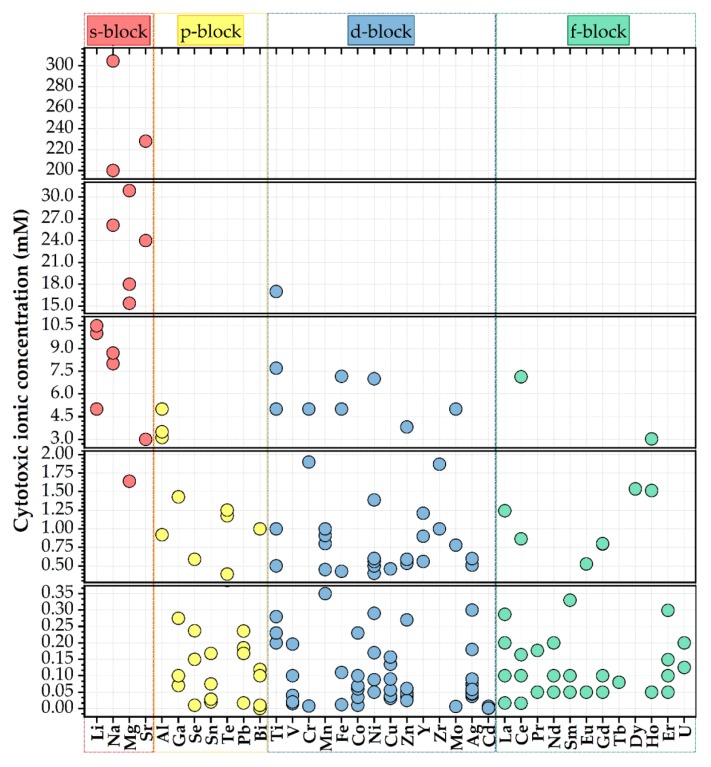
Half-maximal cytotoxic concentration (in mM) of various cationic species [36].

Rare earth-based materials present important, valuable benefits relative to other existing photoluminescent materials as result of their photostabilities, inferior toxicities (Figure 6), great thermal and chemical stabilities, sharp emission bands, and high luminescence quantum yields [47].

### 2.1. Hydroxyapatite Doped with Terbium

Terbium is an essential element of the rare earth family that presents complex optical, magnetic, and electronic properties. It is also an appropriate candidate for applications in the glass industry [48,49], information industry [50], polymers [51,52], bio-chemical sensors [53,54,55], and solar cells [56,57,58]. Terbium is unavoidably present in the environment, organisms, and food chains due to its extensive development and utilization. It is necessary for the human body and/or environment to determine and monitor exposure to Tb^3+^ because of its increased discharge of toxic properties and other adverse effects [50]. 

**Figure 7:**
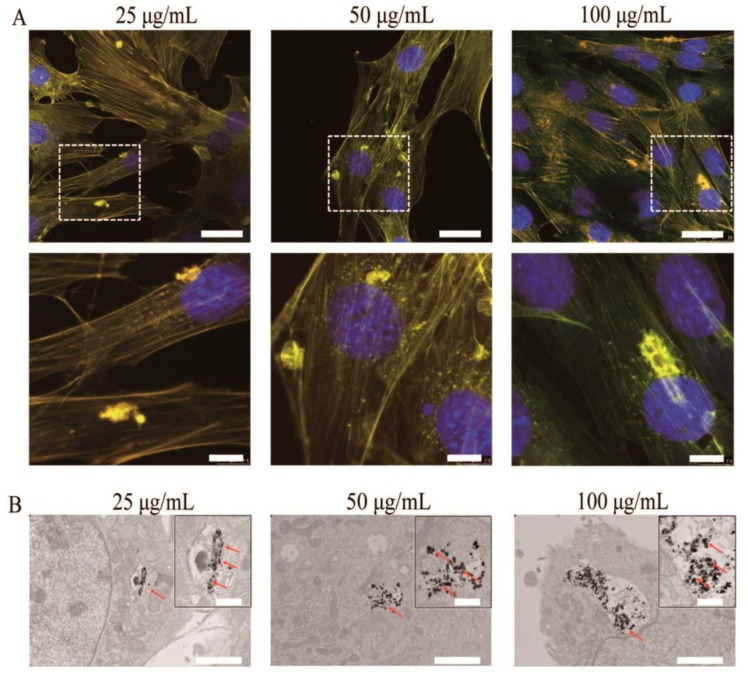
(**A**) Representative fluorescent images of MC3T3-E1 cells after incubation with HA-Tb nanorods for 24 h at 37 °C. (**B**) Transmission electron microscopy images of MC3T3-E1 cells after incubation with HA-Tb nanorods.

Li et al. [37] proposed an appropriate approach to synthesize nano-hydroxyapatite, whose luminescent properties could be stimulated under visible excitation. The method replaces the surface calcium ions on hydroxyapatite nanoparticles with a specific quantity of Tb^3+^. They prepared an inorganic biological probe by doping 20 nm hydroxyapatite with terbium ions. The calcium ions on the hydroxyapatite particle’s surface can be partially substituted by terbium. Nevertheless, a very low amount of the doped Tb^3+^ ions on the hydroxyapatite surface can considerably increase its luminescence, maintaining, at the same time, the principal physicochemical properties and bioactivity of hydroxyapatite. The nanoparticles of the terbium-doped hydroxyapatite were obtained at room temperature, and the atomic ratio of Tb:(Ca + Tb) was 2:100. It was proved experimentally that the Tb-hydroxyapatite nanoparticles could induce a constant luminescence. Moreover, these fluorescent Tb-HA nanoparticles can be incorporated by living cells. Even if the total number of Tb^3+^ ions in the material is moderate, it should be noted that the terbium is nearly nontoxic, because its lethal dose is 50 g via an oral route (LD50PO), which is more than 5 g/kg of terbium nitrate in rats. Using a visible light beam (488 nm), the green emission of the hydroxyapatite nanoparticles could be stimulated. The ready internalization of the nanoparticles and its steady fluorescence indicate that the terbium ion-doped hydroxyapatite represents an excellent inorganic probe for living cells with high biocompatibility. After incubation with rabbit bone marrow mesenchymal stem cells (MSCs) in culture, the luminescent hydroxyapatite was clearly visible under a fluorescent microscope. It was determined that the obtained terbium-doped hydroxyapatite could represent a promising biological probe for cellular research. These results indicate that the Tb-doped HA has great potential to advance the development of luminescent nanoparticles, which is an important subject in nanobiotechnology and medicine. Moreover, in order to evaluate the potential applications of HA-Tb^3+^ in the biomedical field, studies on the biological reaction of MC3T3-E1 cells with different concentrations of Tb^3+^-doped hydroxyapatite (HA-Tb) nanorods (25, 50, and 100 μg/mL) were performed [59]. The photoluminescence of the internalized hydroxyapatite in the intracellular cytoplasm was visible under a fluorescent microscope, and the cells retained their natural morphology (Figure 7).

**Figure 8:**
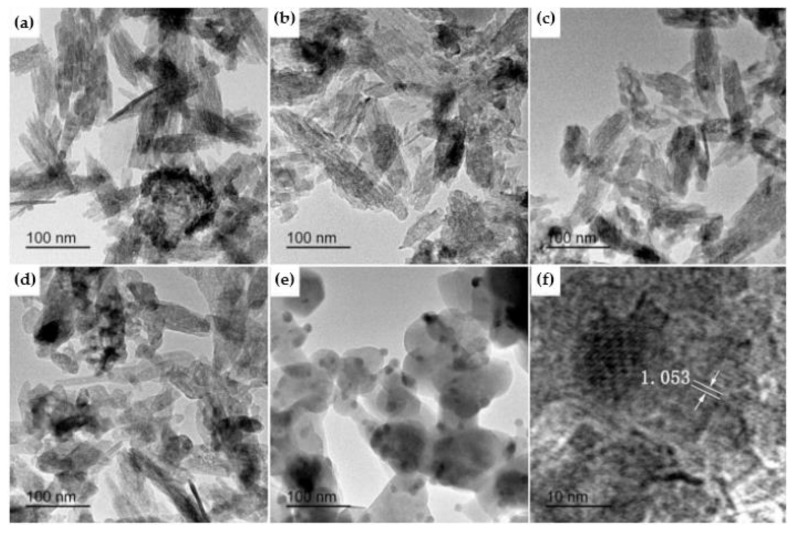
TEM of 6 mol % Tb-HA calcined at different temperatures: (**a**) 300 °C, (**b**) 400 °C, (**c**) 500 °C, (**d**) 600 °C, and (**e**) 700 °C; (**f**) HRTEM image of Tb-HA calcined at 700 °C [61].

Wang et al. [60] published a study in which luminescent Tb-doped hydroxyapatite nanoparticles were obtained via a microemulsion-mediated solvothermal process. The raw materials used for the synthesis were calcium nitrite, terbium oxide, and diammonium hydrogen phosphate. The authors demonstrated that the resulting Tb-doped hydroxyapatite nanoparticles presented homogeneous distribution with two different morphologies (rod- and sphere-like) and notable luminescent properties. A study published by Hairong Yin et al. [61] in 2017 also presented a similar morphological aspect of Tb-HA. As observed from Figure 8, the resulting Tb-HA powders had needle-like morphology at lower temperature, but with the increase of calcination temperature, the shape changed to short rod-like and sphere-like. In addition, the evident excited peak could be identified under a 400-nm wavelength, which promotes Tb-HA as fluorescent bio-probe [60].

The Commission International del’Eclairage coordinates were useful in the determination of the exact emission color and color purity of the samples [61]. 

**Figure 9:**
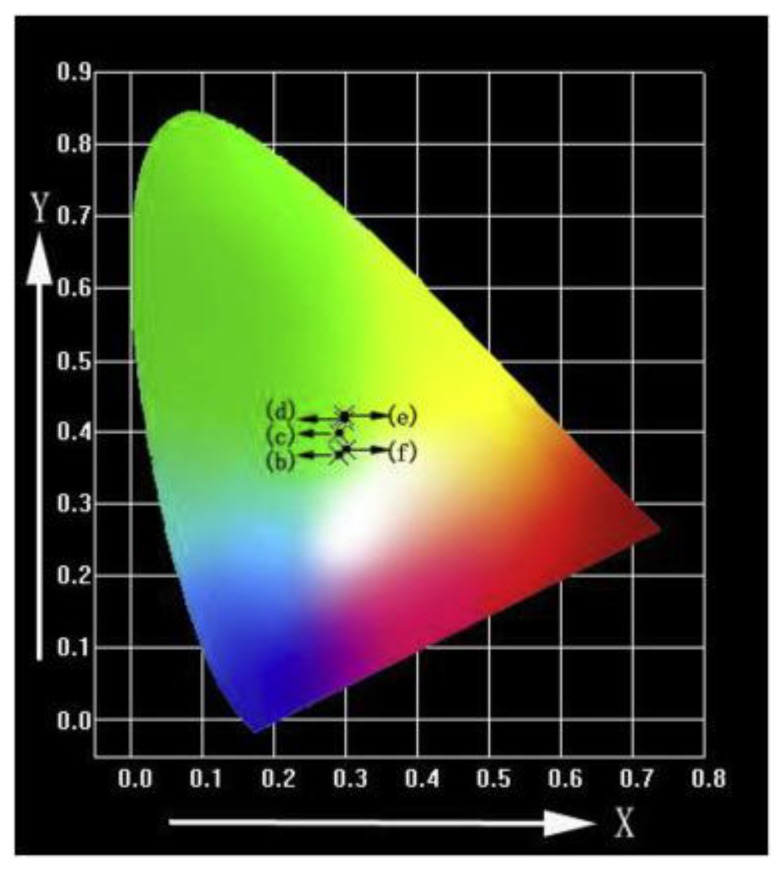
CIE (Commission International del’Eclairage) chromaticity diagram of 6 mol % Tb-HA determined by the corresponding emission spectra under excitation at λ_ex_ = 378 nm [61].

Qiao et al. [62] prepared hydroxyapatite particles doped with Tb^3+^ via a chemical deposition method. The x-ray diffraction (XRD) and Fourier-transform infrared spectroscopy (FTIR) results showed that Tb^3+^ doping has no important influence on the structure of hydroxyapatite. The photoluminescent spectra of the Tb-HA samples demonstrated that the finest excitation light is 378 nm when the wavelength of the monitoring light is 545 nm. When the doping mole fraction of Tb^3+^ is 8%, the luminescent intensity of the Tb-HA sample reaches the maximum. Moreover, the fluorescent lifetime of the Tb-hydroxyapatite samples show a decreasing trend along with the increasing of the Tb^3+^ concentration. The green emission spectra of 6 mol % Tb-HA determined by their corresponding emission spectra under excitation at λ_ex_ = 378 nm was noticed by Hairong Yin et al. [61] (Figure 9 and Table 2).

Terbium ions can be used as a probing reagent to mapping metal-binding sites on valuable biological macromolecules by reason of its comparable chemical characteristics to calcium (Ca^2+^) or magnesium (Mg^2+^) ions. Generally, the luminescent properties of terbium complexes represent the support of many fluorescence applications in clinical chemistry and molecular biology on the grounds of the intramolecular energy transfer through the triplet state of the ligand to the emitting level of the Tb ion [2].

### 2.2. Hydroxyapatite Doped with Erbium

The existence of erbium (Er^3+^) in rib bones has been studied, and it is supposed that the doping of hydroxyapatite with erbium ions could improve the biological properties of hydroxyapatite. Er_2_O_3_ may be exploited to dope calcium phosphate with Er^3+^ ions in order to stipulate photo sites for absorption laser energy, which may contribute to the densification of the manufactured enamel mineral over typical dentine. Moreover, the photoluminescent properties of Er^3+^ can be used to study the in vivo resorption of Er-HA, which can be applied to supervise the bone healing process [9].

Alshemary et al. [9] prepared nano-sized erbium-doped hydroxyapatite using a microwave-assisted precipitation method. It was observed that the crystallinity and the particle dimensions reduced from 75% and 78 nm to 36% and 25 nm, respectively, with the growth of the Er^3+^ amount for doping hydroxyapatite. The photoluminescence test revealed the existence of red and green emission in the spectra. The study showed that Er-HA could be a promising alternative as a sensing material in the future. In vitro bioactivity tests for erbium-doped hydroxyapatite materials conducted in simulated body fluid (SBF) revealed the production of an apatite layer with a (Ca + Er)/P ratio in the range of 1.59–1.72. For that reason, Er-HA can be designed as a possible biomedical material. Alshemary et al. [9] also described a promising method to obtain mesoporous erbium-doped hydroxyapatite by applying a microwave-assisted wet precipitation approach. They obtained results that showed the production of crystalline Er-HA with a crystallite size of 25 nm and a spherical and rod-like morphology. The transmission electron microscopy (TEM) analysis proved the mesoporous structure of the obtained particles. 

The optical spectra of erbium-doped HA consist of seven electron transitions, although a blue shift in the energy band gap (Eg) was noticed upon the increase of the Er^3+^ amount. The photoluminescence spectra consisted of green and red emissions [9]. An in vitro bioactivity test performed in simulated body fluid (SBF) showed that the substitution of Ca^2+^ with Er^3+^ ions into the hydroxyapatite structure leads to a rapid discharge of Er^3+^ ions, showing a strong increase of apatite grains on the surface of the Er-HA pellets with a Ca/P ratio of 1.72 [9].

Pham et al. [24] synthesized erbium-doped hydroxyapatite using a coprecipitation method. The near-infrared luminescence emission of hydroxyapatite may be obtained definitely by doping with erbium. The photoluminescent properties of the erbium-doped hydroxyapatite utilizing a thermal annealing treatment (=1100–1200 °C) were demonstrated by a strong band emission at ~1540 nm, which was more intense than those of the lower-temperature-annealed erbium-doped hydroxyapatite. This improvement of the photoluminescence was, for the most part, associated with the incorporated β-TCP (tricalcium phosphate) phase in the material’s microstructure, which is an inhibitor of photo quenching. Erbium-doped hydroxyapatite can be used as potential material in applications such as waveguide telecommunication and biomedicine [24].

Erbium represents an appropriate element for doping the materials designed for luminescent and biocompatible materials because of its light emission spectra and great biocompatibility [24].

### 2.3. Hydroxyapatite Doped with Europium

Lanthanide ions, such as europium or terbium, present regular fluorescence for cell imaging under a specific excitation wavelength. Particularly, doping with europium, which has 4f–4f intraorbital electronic transitions covering the visible and near-infrared ranges, leads to a relevant material for biomedical applications in the near-infrared spectral range [38]. 

Escudero [47] synthesized europium-doped calcium hydroxyapatite nanoparticles using a microwave-assisted approach. An aqueous solution with a stoichiometric amount of sodium phosphate monobasic was combined with another aqueous solution consisting of calcium nitrate tetrahydrate, europium-(III) nitrate pentahydrate, and polyacrylic acid. The final concentration of calcium, phosphate, and poly(acrylic acid) were 0.06 mol dm^−3^, 0.036 mol dm^−3^, and 2 mg cm^−3^, respectively. The solution’s pH was adjusted to 11 by adding aqueous ammonia. Due to their luminescent properties (red luminescence), their small toxicities (negligible toxicity for Vero cells), and the functionalization with poly(acrylic acid), which induces the capacity for further functionalization with molecules of biomedical interest, these nanophosphors appear to be a promising candidate for use as potential tools for biomedical applications. 

**Figure 10:**
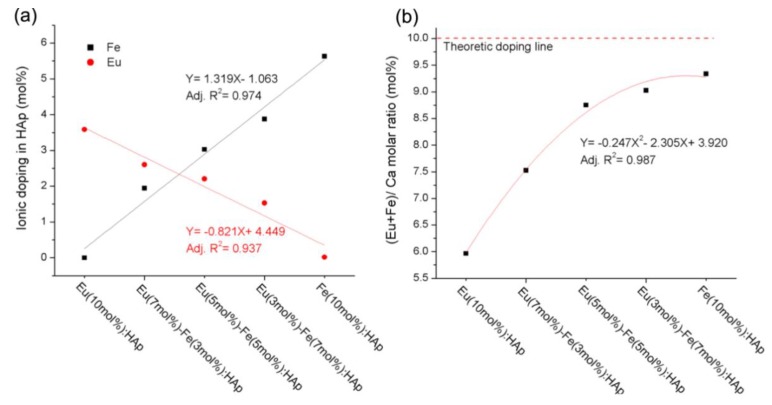
The ICP-AES (Inductively Coupled Plasma Atomic Emission Spectroscopy) results: (**a**) Eu^3+^ and Fe^3+^ doping amount; (**b**) (Eu + Fe)/Ca molar ratio of hydroxyapatite with different dopant concentrations [38].

Chen et al. [38] reported a study on the doping mechanism and photoluminescence of Eu/Fe:HA nanoparticles. The luminescent Eu/Fe:HA nanoparticles were synthetized via wet chemical precipitation in water, without the addition of any surfactant. The doping concentrations of Eu^3+^ and Fe^3+^ in hydroxyapatite measured by ICP-AES (Inductively Coupled Plasma Atomic Emission Spectroscopy) were similar to the theoretical values (Figure 10).

**Figure 11:**
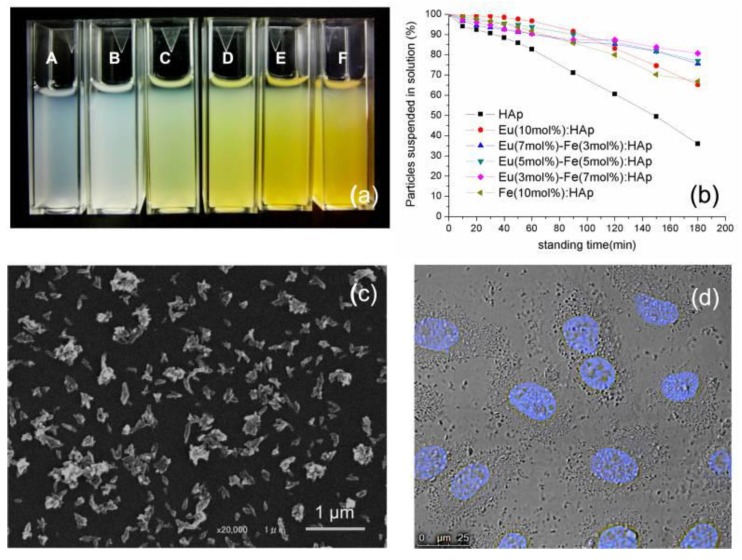
(**a**) Re-suspended HA and Eu/Fe:HA particles in a culture medium with 10% FBS (fetal bovine serum)-A-HA; molar ratios of Eu^3+^ to Fe^3+^ from B to F: 1:0, 2:1, 1:1, 1:2, and 0:1. (**b**) Assessment of particle suspension in a culture medium by turbidity measurement at 320 nm. (**c**) SEM image of Eu (5 mol %)-Fe (5 mol %):HAp. (**d**) cells incubated with Eu (5 mol %)-Fe (5 mol %):HA; (cell nucleus—Hoechest stain) [38].

**Figure 12:**
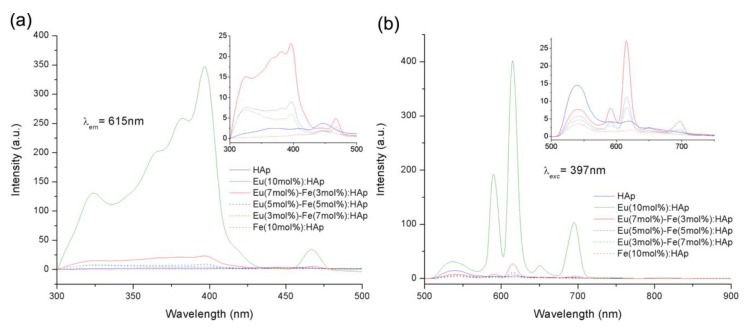
(**a**) The PL (photoluminescence) excitation spectra and (**b**) PL emission spectra [38].

The Eu/Fe:HA single-phase nanoparticles were situated in the nanometer domain, with dimensions of about 100 nm and rod-like morphology. Due to their biological properties, Eu/Fe:HA particles could be suspended in a culture medium (Figure 11). The nanoparticles were obtained as hexagonal single crystals without the presence of a second phase, and the nanoparticles belonged to the biodegradable, B-type, carbonated hydroxyapatite. These synthetized nanoparticles present luminescence at notable peaks at 536, 590, 615, 650, and 695 nm under 397 nm excitation (Figure 12). The results show that Eu/Fe:HA nanoparticles could be a perfect candidate for biological applications [38]. 

Yang et al. [63] prepared luminescent, bioactive, and mesoporous europium-doped hydroxyapatite (Eu-HA) via a simple one-step method, using cationic surfactant as a template. The prepared multifunctional hydroxyapatite was used as a drug delivery carrier in order to examine the drug storage and/or release properties. The obtained results displayed the typical ordered characteristics of the hexagonal mesostructure, with rod-like morphology and a particle size of 20–40 nm in diameter and 100–200 nm in length.

The drug storage and release assay showed that the luminescent hydroxyapatite presents a very similar drug loading amount and cumulative release rate to those of the pure hydroxyapatite. The ibuprofen-loaded samples still showed the red luminescence of Eu^3+^ (^5^D_0_–^7^F_1_,_2_) under UV irradiation, and the emission intensities of Eu^3+^ in the drug carrier system differed with the released amount of ibuprofen, thus making the drug release easy to follow and control by varying the intensity of the luminescence. This system exhibited a potential application in the area of drug delivery and disease therapy due to its bioactive, luminescent and mesoporous properties [63].

Han et al. [64] presented a cell labeling approach utilizing biocompatible, europium-doped hydroxyapatite (Eu-HA), inorganic nanoparticles. Eu-HA nanoparticles were obtained via a precipitation method and used to label Bel-7402 human liver cancer cells as a fluorescent probe. The europium-doped hydroxyapatite nanoparticles were internalized by the cells. After that, the strong green and red fluorescence were visible by the irradiation of blue and green light, respectively. The Eu-HA nanoparticles presented a temporally stable luminescence and could be excited by wavelengths in the visible area. The result demonstrated that the Eu-HA nanoparticles represent a potential material for use in the field of biocompatible fluorescent labeling in biological studies [64]. 

A paper published by Yang et al. [65] proposed the synthesis of Eu^3+^ and Tb^3+^-doped hydroxyapatite phosphors with homogeneous morphology and narrow size distribution through a CTAB (Cetyltrimethylammonium bromide) /water/*n*-octane/*n*-butanol microemulsion process under hydrothermal treatment. The prepared Eu-HA and Tb:HA phosphors presented the typical emission lines of Eu^3+^ and Tb^3+^, and the decay curves of both samples were appropriate with a double-exponential function. 

In addition, the photoluminescence intensities of these samples increased with the solvothermal temperature as a result of the enhanced crystallinity. The photoluminescence emission intensities of Eu^3+^ and Tb^3+^ increased with the rise of their concentration and reach a maximum at 5 mol % concentration of Ca^2+^ and then decreased with further increased concentration, which could be a consequence of the concentration quenching effect. The decrease of the fluorescence intensity with further increased concentration can also be caused by self-absorption.

These phosphors show promise as candidates for carriers for drug release and targeting due to their biocompatible and luminescent properties [65].

A study published by Doat et al. [66] reported a novel inorganic bio-probe synthetized at a low temperature (37 °C). The bio-probe consisted of mineral nanoparticles of apatite tricalcium phosphate doped with europium. The size, structure, and composition of the prepared material were similar to those of the mineral part of the calcified tissues. The red luminescence of the obtained probe was photostable, in contrast to organic probes, which degrade immediately due to photobleaching. This luminescence could be obtained under visible irradiation, which makes it appropriate for extended examinations of living cells. For this purpose, human pancreatic epithelial cells in culture were incubated in the presence of these particles, and their internalization could be detected by laser scanning confocal microscopy. 

Transmission electron microscopy and electron microdiffraction analysis proved that the particles were internalized while maintaining their apatite structure. This probe appears to be a promising approach for biovectorization [66].

According to presented studies, Eu^3+^ is quite a suitable dopant ion for hydroxyapatite because of the ease with which it incorporates into the HA crystal lattice as a result of its similar ionic radius. Furthermore, Eu^3+^ displays excellent optical properties and emits in the visible region, exhibiting mostly red luminescence with a high Stokes shift that simplifies excitation and subsequent detection [67].

### 2.4. Hydroxyapatite Doped with Lanthanum

La^3+^-substituted hydroxyapatite displays excellent biocompatibility compared with pure hydroxyapatite. A few studies have been conducted in order to demonstrate the benefits of lanthanum in the biomedical field.

Mayer et al. [68] obtained hydroxyapatites by precipitation from an aqueous solution with La^3+^ (0–0.75%) and carbonate (0–6.1%) at a controlled pH of 7.0. The assimilation of La^3+^ was 90–95% complete. Due to nonstoichiometry, the Ca/P (1.54–1.63) ratios were relatively low. With the aid of IR spectroscopy, it was determined that the carbonate from the samples was a B-type carbonate. The lattice parameters of the hexagonal apatite structure were not changed because of the La^3+^ amount. 

After heating to 800 °C, the noncarbonated samples were partially transformed into β-Ca_3_(PO_4_)_2_. Thermogravimetric analysis indicated a release of 0.4 mol adsorbed and 1 mol crystalline water up to 400 °C and the deterioration of the carbonate up to 900 °C in the obtained samples. These results reveal that La^3+^ could replace Ca^2+^ in the crystal lattice of hydroxyapatite [68].

Phase pure hydroxyapatite and lanthanum phosphate powders were obtained by Ghosh et al. [69] using a wet chemical synthesis method. Composite samples were obtained by mixing the powders, pressing, and sintering. 

The composite nature of the prepared pellets incorporating up to 50 wt.% lanthanum phosphate (H0 − 100%Hap, HL1 − 90% Hap + 10%LP, HL2 − 80% Hap + 20%LP, HL3 − 70% Hap + 30%LP, HL4 − 600% Hap + 40%LP, HL5 − 50% Hap + 50%LP) was achieved even up to sintering at 1150 °C. A continuous decline in densification, bending strength, and hardness were reached by increasing the content of the lanthanum phosphate. It was found that the obtained composites could be drilled by solid carbide drill bits up to 1150 °C. The composites with a high lanthanum phosphate amount presented better processability compared with the ones with a lower lanthanum phosphate content, and also the force and torque required was much higher. The materials sintered at 1200 °C revealed a new phase. Thus, the composite structure was not maintained, and the drillability was also not obtained for any amount of the lanthanum phosphate content. All the different prepared compositions (H0, H1, H2, H3, H4, H5) demonstrated positive character for in vitro bioactivity and biocompatibility assay. The cell viability also displayed the irrelevant difference in the results with the increasing lanthanum phosphate amount [69]. 

**Figure 13:**
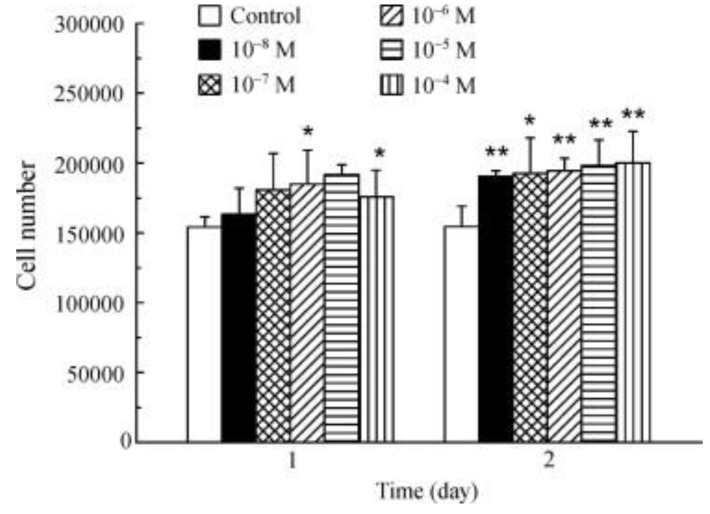
Effect of La^3+^ on osteoblasts proliferation (* *p* < 0.05; ** *p* < 0.01) [70].

La^3+^ promotes osteoblast proliferation (Figure 13). The incubation of osteoblasts with La^3+^ (1 × 10^−8^–1 × 10^−4^ M) showed that all the tested concentrations significantly increased the cell number after 2 days [70].

Jadalannagari et al. [71] synthesized rod-shaped nanoparticles of lanthanum-doped hydroxyapatite (La-HA) using a modified sol–gel method at a low temperature of 100 °C. The (La + Ca):P proportion was maintained at 1.67 while the La/(Ca + La) ratio was varied as *x* = 0.02, 0.06, 0.1. By increasing the concentration of La from *x* = 0.02 to 0.1 an increase in crystallinity and crystallite size was observed. These obtained particles were internalized by human embryonic kidney and human adenocarcinoma cells and showed fluorescence when detected under tetramethylrhodamine (TRITC) and fluorescein (FITC) filters using epifluorescence microscopy. Lanthanum-doped hydroxyapatite nanoparticles with *x* = 0.02 displayed the highest internalization, maximum intracellular fluorescence, and no cytotoxicity. It was observed that the cell viability and internalization decreased with increases in the La amount. Nanoparticles corresponding to *x* = 0.1 presented spindle-shaped morphology [71].

Nano-rods of pure hydroxyapatite and lanthanum-doped hydroxyapatite were obtained by Ahymah [72] using a sol–gel route and calcium nitrate and diammonium hydrogen phosphate as precursors. The samples did not contain La_2_O_3_ or β-TCP phases, compared with the samples obtained by the solid state and co-precipitation methods. The control sample was noted as CHAp, and the lanthanum-doped samples with 10 mM, 20 mM, 30 mM, 40 mM, and 50 mM were denoted as L1HAp, L2HAp, L3HAp, L4Hap, and L5HAp. Enhancement in the amount of lanthanum resulted in the increase of the crystallinity and crystallite size. Furthermore, enhancements in the specific surface area (31%) and hardness (14%) were noticed in the doped samples. The tests using phosphate-buffered saline (PBS) showed a reduction in the dissolution of the samples as a result of the enhancement in the dopant concentration. 

An in vitro study of drug release was determined by measuring the absorbance using a UV spectrophotometer at 230 nm. The hydroxyapatite sample and amoxicillin was taken in 2:1 ratio, and it was mixed thoroughly and made into a pellet. The bactericidal effect against Gram positive and Gram negative bacteria showed high antimicrobial resistant activity of the samples when they were functionalized with amoxicillin [72].

La^3+^-doped hydroxyapatite is a promising approach in the biomedical field for biocompatible fluorescent probes applied in cellular internalization and drug releasing agents over a long period of time.

### 2.5. Hydroxyapatite Doped with Dysprosium

Dysprosium is considered to be a heavy rare earth element (HREE). Dysprosium-165, its radioactive isotope, was studied for potential applications in the field of medicine. Radiation with dysprosium-165 has been demonstrated to be more efficient in treating injured joints than traditional treatment and surgery.

Dysprosium-doped hydroxyapatite represents an attractive option for a biocompatible ceramic material for luminescence imaging applications.

Tesch et al. [67] prepared luminescent and magnetic hydroxyapatites by doping with europium (Eu^3+^) and dysprosium (Dy^3+^). A co-doping of Eu^3+^ and Dy^3+^ was used in order to blend the requested physical properties. Both REE ions, Eu^3+^ and Dy^3+^, were assimilated into the hydroxyapatite crystal lattice, where they preferentially chose calcium sites. Eu-doped hydroxyapatite presents a dopant concentration with persistent photoluminescent properties, while Dy-doped hydroxyapatite exhibits paramagnetic comportment as result of the high magnetic moment of Dy^3+^. Co-doped HA nanoparticles blend both properties into one single crystal. Curiously, the multimodal co-doped hydroxyapatite increased its photoluminescent properties due to the energy transfer from the Dy^3+^ sensitizer to the Eu^3+^ activator ions. Eu:Dy:HA presents strong transverse relaxation effects with a maximum transverse relativity of 83.3 L/(mmol·s). Because of their tunable, photoluminescent, magnetic properties and cytocompatibility Eu-HA, Dy:HA, and Eu:Dy:HA serve as alternative biocompatible ceramic materials with applications in luminescence imaging and may also be suitable as a contrast agent for magnetic resonance imaging (MRI) in implantology or functional coatings [67]. 

A study published by Sánchez et al. [6] in 2015 evaluated the toxicity of hydroxyapatite doped with dysprosium cations and hydroxyapatite loaded with folic or glucuronic acids. Nanoparticles functionalized with folic acid are able to identify cancer cells. Functionalization with glucuronic acid decreases the toxicity levels, because this acid helps to eliminate nanoparticles through urine. Hydroxyapatite doped with dysprosium was synthesized by co-precipitation. This modified structure revealed fluorescent character, which can be identified with a fluorometer, stimulated at 344 or 360 nm by confocal microscopy. The contrast effect in MRI images at a concentration of 0.2 mg mL^−1^, considering the effect on the transversal relaxation time of protons in water, represents an important feature of these particles. The biochemical tests indicated that the increase of oxidative stress indicators (lipoperoxides (LPO) and nitric oxides) generated by hydroxyapatite and hydroxyapatite-Dy particles present in the kidney, lungs, and liver could be constricted by functionalizing these particles with glucuronic or folic acid. The increase of ATP activity caused by hydroxyapatite can be reduced by adopting the substituted forms, HA-Dy, HA-F, and HA-G, respectively, at doses of 10 mg, and the HA particles’ toxicity could be decreased by functionalizing with folic or glucuronic acid [6].

## 3. Conclusions

As the major inorganic constituent of human bone and tooth enamel, hydroxyapatite presents highly biocompatible and bioactive properties. 

Pure hydroxyapatite nanoparticles do not possess luminescent properties. As a consequence, they are complicated to identify in living cells during in vitro tests.

In the study of biological processes in living cells, it is necessary to examine all the modifications that can occur in situ. Biological probes are extensively used as the most competent instruments for biological staining and diagnostics.

Considering its proper bioactive, biocompatible, osteoconductive, biodegradable, and nonimmunogenic properties, HA can play an extraordinary role in the areas of tissue engineering, drug release, and gene delivery and in different biomedical fields. At the same time, hydroxyapatite nanocrystals are promising candidates for host materials for rare earth element doping, which could provide novel fluorescent properties. These lanthanide-doped hydroxyapatite nanoparticles could be utilized as photoluminescent probes in biological imaging, considering that they not only maintain their great biocompatibility and valuable biodegradability, but also present more improved features than other developed optical probes and have been successfully applied in the fluorescence imaging of cells (Table 3).

The photoluminescent hydroxyapatite nanoparticles utilized in biological probes are generally prepared in an aqueous solution; they can easily agglomerate and can even present flaws in crystallinity or uniformity sizes. Nevertheless, the fluorescence and imaging quality of rare earth ion-doped hydroxyapatite nanoparticles are altered by size, crystallinity, size distribution, and the dispersed state of the nanocrystals.

Lanthanide ions have been extensively used as luminescent sensors due to their long excited-state lifetimes, sharp emission bands, large Stokes’ shifts, and sensitive and efficient luminescence detection because of their capacity to remove the interference from the fast decaying background fluorescence.

Hydroxyapatite doped with rare earth elements presents enhanced biological properties. La^3+^-substituted hydroxyapatite displays great biocompatibility when compared with pure hydroxyapatite. It also constrains the spread of dental caries, serves as a promising biocompatible fluorescent probe for cellular internalization, and is a good drug-releasing agent for a long period of time. At the same time, Eu^3+^-doped hydroxyapatite is a valuable drug carrier, which can be quickly detected in vivo because of its notable photoluminescent emission. 

Dy^3+^-doped hydroxyapatite also represents a promising biocompatible ceramic material for luminescence imaging. Tb^3+^-doped hydroxyapatite nanoparticles may also be used in order to obtain an inorganic florescent probe with superior biocompatibility properties and stability in cells. The doping of hydroxyapatite with Er^3+^ may improve the biological characteristics of hydroxyapatite and could induce enhanced photoluminescent properties.

## Figures and Tables

**Table 1:** Synopsis of the realm of bio-functionality of cation-substituted hydroxyapatites (Has) [36].

Cation (M)	Sample Form	Doping Range {M/(M + Ca)}100 (at %)	Bio-Functionality/Effect of the Dopant
Tb	Powder	2–17	In vitro cytocompatibility with MC3T3-E1 (doses of 25–100 µg mL^−1^ Tb-HA-NPs) and A549 (doses of 20–320 µg mL^−1^ Tb-HA-NPs) cell lines.
Er	Powder	2–10	Induces the formation of biomimetic apatite in-growths in simulated body fluid (SBF).
Eu	Powder	0.1–20	Induces the in vitro formation of bone-like apatite in SBF; In vitro cytocompatibility with MG-63 (cell proliferation up to 4 days), HeLa, human embryonic kidney HEK 293, L929 (viability >80% for Eu-HA doses of 25–500 µg mL^−1^); Low cytotoxicity for human gingival fibroblast (HGF-1) cells after 24 h (500–2000 µg mL^−1^); Cytotoxicity for transformed human umbilical vein endothelial cells (T-HUVEC) after treatment with 0.3–30 µg mL−1 of 5 at % doped HA; Ability to kill cervical HeLa cells after 24 h when combined with 5 fluorouracil (5FU); Negligible toxicity by hen’s egg test on the chick area vasculosa (HET-CAV); Antibacterial effect against *E. faecalis* * (ATCC 29212), *S. aureus* * (0364), and *P. aeruginosa* * (1397); No antibacterial activity against *E. coli* * even at high doping; Antifungal effect against *C. albicans* * (ATCC 10231) with a doping content of 20 at %.
La	Powder Coating	2–30	In vitro cytocompatibility with MC3T3-E1 and L929 cell lines; No cytotoxicity for adenocarcinoma (MCF-7) and human embryonic kidney HEK cells at a doping level of 2 at %; Antibacterial effect against *S. aureus* (e.g., ATCC 25175), *E. coli*, *P. aeruginosa*, and *Bacillus*; Improvement of mechanical properties: bonding strength and Vickers hardness.
Dy	Powder	0.5–10	In vitro cytocompatibility with L929 cell line; Negligible toxicity by hen’s egg test on the chick area vasculosa (HET-CAV); Increase of oxidative stress lipoperoxides and nitric oxide indicators in the kidney, lungs, and liver of rats; lower activity of anti-oxidant glutathione peroxidase enzyme.

* *E. faecalis*—Enterococcus faecalis, *S. aureus*—Staphylococcus aureus, *P. aeruginosa*—Pseudomonas aeruginosa, *E. coli*—Escherichia coli, *C. albicans*—Candida albicans.

**Table 2:** Commission International del’Eclairage (CIE) coordinates of 6 mol % Tb-HA [61].

Samples	(b) 300 °C	(c) 400 °C	(d) 500 °C	(e) 600 °C	(f) 700 °C
X	0.2893	0.2910	0.2981	0.2985	0.3000
Y	0.3682	0.3976	0.4161	0.4234	0.3743

**Table 3:** Influence of different doping elements on HA properties and the biomedical applications.

Doping Element	Synthesis Method	Improvements of Photoluminescent Properties	Biomedical Application	References
Terbium	microemulsion-mediated solvothermal process	the particles could be excited by a visible light beam at 400 nm	fluorescent bio-probe	Wang et al., 2010 [60]
	chemical deposition	excitation light is 378 nm when the wavelength of the monitoring light is 545 nm	fluorescent probe	Qiao et al., 2015 [62]
Erbium	microwave-assisted precipitation method	red and green emission in the spectra	sensing material	Alshemary et al., 2015 [9]
	Microwave-assisted wet precipitation	photoluminescence spectra—green and red emissions	bone healing process	Alshemary et al., 2015 [9]
	co-precipitation	near-infrared emission peaks ~1540 nm	biomedicine	Pham et al., 2016 [24]
Europium	microwave-assisted synthesis	red luminescence; negligible toxicity for Vero cells	potential tools for biomedical applications	Escudero, 2013 [47]
	wet chemical precipitation in water without the addition of any surfactant	luminescence at peaks at 536, 590, 615, 650, and 695 nm under 397 nm excitation	fluorescent probe for in vivo imaging	Chen et al., 2014 [38]
	simple one-step method using cationic surfactant as a template	red luminescence of Eu^3+^ (^5^D_0_–^7^F_1,2_) under UV irradiation	drug delivery disease therapy	Yang et al., 2008 [63]
	precipitation	strong green and red fluorescence by irradiation of blue and green light	biocompatible fluorescent labeling material in biological studies	Han et al., 2010 [64]
	synthetized at low temperatures (37 °C)	red luminescence is photostable; luminescence could be obtained under visible irradiation	bio-probe	Doat et al., 2003 [66]
Europium and Terbium	microemulsion process under hydrothermal treatment	typical emission lines of Eu^3+^ and Tb^3+^	carriers for drug release and targeting	Yang et al., 2008 [65]
Lanthanum	wet chemical synthesis method	in vitro bioactivity and biocompatibility	bioimaging phosphor/luminescent labeling materials for bioimaging	Ghosh et al., 2016 [69]
	modified sol–gel method at a low temperature of 100 °C	fluorescence detected under TRITC (Tetramethylrhodamine) and FITC (Fluorescein isothiocyanate) filters using epifluorescence microscopy	fluorescent probes for cellular internalization and biolabeling	Jadalannagari et al., 2014 [71]
	sol–gel route	decrease in the dissolution of the samples as the dopant concentration increases	implant in biomedical field	Ahymah, 2011 [72]
Dysprosium and Europium	co-doping	increased photoluminescent properties; strong transverse relaxation effects	contrast agent for MRI in implantology or functional coatings	Tesch et al., 2017 [67]
Dysprosium	co-precipitation	fluorescent character—stimulated at 344 or 360 nm	bimodal probes with low toxicity	Sánchez et al., 2015 [6]

## References

[1] Qi S., Huang Y., Li Y., Cai P., Kim S.I., Seo H.J. (2014). Probe spectrum measurements of Eu^3+^ ions as a relevant tool for monitoring in vitro hydroxyapatite formation in a new borate biomaterial. J. Mater. Chem. B.

[2] Zhang C., Li C., Huang S., Hou Z., Cheng Z., Yang P., Peng C., Lin J. (2010). Self-activated luminescent and mesoporous strontium hydroxyapatite nanorods for drug delivery. Biomaterials.

[3] Bach L.G., Cao X.T., Islam M., Kim H.G., Lim K.T. (2015). Combination of surface initiated reversible addition fragmentation chain transfer polymerization, thiol-ene click chemistry and coordination chemistry for the fabrication of a novel photoluminescent hydroxyapatite nanohybrids. J. Nanosci. Nanotechnol..

[4] Liu H., Chen F., Xi P., Chen B., Huang L., Cheng J., Shao C., Wang J., Bai D., Zeng Z. (2011). Biocompatible Fluorescent Hydroxyapatite: Synthesis and Live Cell Imaging Applications. J. Phys. Chem. C.

[5] Huang K., Martí A.A. (2012). Optimizing the Sensitivity of Photoluminescent Probes Using Time-Resolved Spectroscopy: A Molecular Beacon Case Study. Anal. Chem..

[6] Sánchez Lafarga A.K., Pacheco Moisés F.P., Gurinov A., Ortiz G.G., Carbajal Arízaga G.G. (2015). Dual responsive dysprosium-doped hydroxyapatite particles and toxicity reduction after functionalization with folic and glucuronic acids. Mater. Sci. Eng. C.

[7] Fu D.-L., Jiang Q.-H., He F.-M., Yang G.-L., Liu L. (2012). Fluorescence microscopic analysis of bone osseointegration of strontium-substituted hydroxyapatite implants. J. Zhejiang Univ. Sci. B.

[8] Tsai S.-W., Huang S.-S., Yu W.-X., Hsu Y.-W., Hsu F.-Y. (2018). Fabrication and characteristics of porous hydroxyapatite-CaO composite nanofibers for biomedical applications. Nanomaterials.

[9] Alshemary A.Z., Akram M., Goh Y.-F., Abdul Kadir M.R., Abdolahi A., Hussain R. (2015). Structural characterization, optical properties and in vitro bioactivity of mesoporous erbium-doped hydroxyapatite. J. Alloys Compd..

[10] Boanini E., Cassani M., Rubini K., Boga C., Bigi A. (2018). (9R)-9-Hydroxystearate-Functionalized Anticancer Ceramics Promote Loading of Silver Nanoparticles. Nanomaterials.

[11] Dorozhkin S.V. (2009). Calcium Orthophosphates in Nature, Biology and Medicine. Materials.

[12] Liu C., Ji X., Cheng G. (2007). Template synthesis and characterization of highly ordered lamellar hydroxyapatite. Appl. Surf. Sci..

[13] Yang Z., Huang Y., Chen S.-T., Zhao Y.-Q., Li H.-L., Hu Z.-A. (2005). Template synthesis of highly ordered hydroxyapatite nanowire arrays. J. Mater. Sci..

[14] Wang P., Li C., Gong H., Jiang X., Wang H., Li K. (2010). Effects of synthesis conditions on the morphology of hydroxyapatite nanoparticles produced by wet chemical process. Powder Technol..

[15] Liu Y., Hou D., Wang G. (2004). A simple wet chemical synthesis and characterization of hydroxyapatite nanorods. Mater. Chem. Phys..

[16] Madhumathi K., Shalumon K., Rani V.D., Tamura H., Furuike T., Selvamurugan N., Nair S., Jayakumar R. (2009). Wet chemical synthesis of chitosan hydrogel–hydroxyapatite composite membranes for tissue engineering applications. Int. J. Biol. Macromol..

[17] Murugan R., Ramakrishna S. (2005). Crystallographic study of hydroxyapatite bioceramics derived from various sources. Cryst. Growth Des..

[18] Ripamonti U. (1991). The morphogenesis of bone in replicas of porous hydroxyapatite obtained from conversion of calcium carbonate exoskeletons of coral. J. Bone Jt. Surg. Am..

[19] Ripamonti U., Ma S.-S., Reddi A. (1992). Osteogenin, a bone morphogenetic protein, adsorbed on porous hydroxyapatite substrata, induces rapid bone differentiation in calvarial defects of adult primates. Plast. Reconstr. Surg..

[20] Barbeck M., Udeabor S., Lorenz J., Schlee M., Holthaus M.G., Raetscho N., Choukroun J., Sader R., Kirkpatrick C.J., Ghanaati S. (2015). High-temperature sintering of xenogeneic bone substitutes leads to increased multinucleated giant cell formation: In vivo and preliminary clinical results. J. Oral Implantol..

[21] Wei J.-Q., Liu Y., Zhang X.-H., Liang W.-W., Zhou T.-F., Zhang H., Deng X.-L. (2017). Enhanced critical-sized bone defect repair efficiency by combining deproteinized antler cancellous bone and autologous BMSCs. Chin. Chem. Lett..

[22] Arita I., Castano V., Wilkinson D. (1995). Synthesis and processing of hydroxyapatite ceramic tapes with controlled porosity. J. Mater. Sci..

[23] Bhattacharjee B.N., Mishra V.K., Rai S.B., Parkash O., Kumar D. Study of Morphological Behavior of Hydroxyapatite, EDTA Hydroxyapatite and Metal Doped EDTA Hydroxyapatite Synthesized by Chemical Co-Precipitation Method via Hydrothermal Route. Proceedings of the Key Engineering Materials.

[24] Pham V.-H., Van H.N., Tam P.D., Ha H.N.T. (2016). A novel 1540 nm light emission from erbium doped hydroxyapatite/β-tricalcium phosphate through co-precipitation method. Mater. Lett..

[25] Pandi K., Viswanathan N. (2015). In situ precipitation of nano-hydroxyapatite in gelatin polymatrix towards specific fluoride sorption. Int. J. Biol. Macromol..

[26] Ben-Arfa B.A., Salvado I.M.M., Ferreira J.M., Pullar R.C. (2017). Novel route for rapid sol-gel synthesis of hydroxyapatite, avoiding ageing and using fast drying with a 50-fold to 200-fold reduction in process time. Mater. Sci. Eng..

[27] Asri R., Harun W., Hassan M.-A., Ghani S., Buyong Z. (2016). A review of hydroxyapatite-based coating techniques: Sol–gel and electrochemical depositions on biocompatible metals. J. Mech. Behav. Biomed. Mater..

[28] Tilkin R., Regibeau N., Grandfils C., Lambert S. Optimization of hydroxyapatite synthesis via sol-gel process for bone reconstruction application. Proceedings of the 19th international Sol-Gel Conference.

[29] Türk S., Altınsoy I., ÇelebiEfe G., Ipek M., Özacar M., Bindal C. (2017). Microwave–assisted biomimetic synthesis of hydroxyapatite using different sources of calcium. Mater. Sci. Eng..

[30] Pokhrel S. (2018). Hydroxyapatite: Preparation, Properties and Its Biomedical Applications. Adv. Chem. Eng. Sci..

[31] Hu C., Aindow M., Wei M. (2017). Focused ion beam sectioning studies of biomimetic hydroxyapatite coatings on Ti-6Al-4V substrates. Surf. Coat. Technol..

[32] Cho J.S., Lee J.-C., Chung S.H., Seo J.K., Rhee S.-H. (2014). Effect of grain size and density of spray-pyrolyzed hydroxyapatite particles on the sinterability of hydroxyapatite disk. Ceram. Int..

[33] Cho J.S., Lee J.C., Rhee S.H. (2016). Effect of precursor concentration and spray pyrolysis temperature upon hydroxyapatite particle size and density. J. Biomed. Mater. Res..

[34] Zhang M., Liu J.-K., Miao R., Li G.-M., Du Y.-J. (2010). Preparation and characterization of fluorescence probe from assembly hydroxyapatite nanocomposite. Nanoscale Res. Lett..

[35] Schuetz R., Fix D., Schade U., Aziz E.F., Timofeeva N., Weinkamer R., Masic A. (2015). Anisotropy in Bone Demineralization Revealed by Polarized Far-IR Spectroscopy. Molecules.

[36] Tite T., Popa A.-C., Balescu L.M., Bogdan I.M., Pasuk I., Ferreira J.M.F., Stan G.E. (2018). Cationic Substitutions in Hydroxyapatite: Current Status of the Derived Biofunctional Effects and Their In Vitro Interrogation Methods. Materials.

[37] Li L., Liu Y., Tao J., Zhang M., Pan H., Xu X., Tang R. (2008). Surface modification of hydroxyapatite nanocrystallite by a small amount of terbium provides a biocompatible fluorescent probe. J. Phys. Chem. C.

[38] Chen M.-H., Yoshioka T., Ikoma T., Hanagata N., Lin F.-H., Tanaka J. (2014). Photoluminescence and doping mechanism of theranostic Eu^3+^/Fe^3+^ dual-doped hydroxyapatite nanoparticles. Sci. Technol. Adv. Mater..

[39] Chung R.-J. (2011). Study of hydroxyapatite nano composites with photoluminescence properties. Biomed. Eng. Appl. Basis Commun..

[40] Silva C., Sombra A., Rosa I., Leite E., Longo E., Varela J.A. (2008). Study of Structural and Photoluminescent Properties of Ca_8_Eu_2_(PO_4_)_6_O_2_. J. Fluoresc..

[41] Consani S., Balić-Žunić T., Cardinale A.M., Sgroi W., Giuli G., Carbone C. (2018). A Novel Synthesis Routine for Woodwardite and Its Affinity towards Light (La, Ce, Nd) and Heavy (Gd and Y) Rare Earth Elements. Materials.

[42] Boesche N.K., Rogass C., Lubitz C., Brell M., Herrmann S., Mielke C., Tonn S., Appelt O., Altenberger U., Kaufmann H. (2015). Hyperspectral REE (rare earth element) mapping of outcrops—Applications for neodymium detection. Remote Sens..

[43] Iwanaga H. (2010). Emission Properties, Solubility, Thermodynamic Analysis and NMR Studies of Rare-Earth Complexes with Two Different Phosphine Oxides. Materials.

[44] Rodriguez-Ubis J.C., Brunet E., Juanes O. (2013). Lanthanide Ions as Luminescent Probes. Encyclopedia of Metalloproteins.

[45] Hein J.R., Koschinsky A., Mikesell M., Mizell K., Glenn C.R., Wood R. (2016). Marine phosphorites as potential resources for heavy rare earth elements and yttrium. Minerals.

[46] Quarta A., Piccirillo C., Mandriota G., Di Corato R. (2019). Nanoheterostructures (NHS) and Their Applications in Nanomedicine: Focusing on In Vivo Studies. Materials.

[47] Escudero A., Calvo M.E., Rivera-Fernández S., De la Fuente J.M., Ocaña M. (2013). Microwave-assisted synthesis of biocompatible europium-doped calcium hydroxyapatite and fluoroapatite luminescent nanospindles functionalized with poly (acrylic acid). Langmuir.

[48] Deopa N., Rao A. (2018). Spectroscopic studies of single near ultraviolet pumped Tb^3+^ doped Lithium Lead Alumino Borate glasses for green lasers and tricolour w-LEDs. J. Lumin..

[49] Fu Z., Xu P., Yang Y., Li C., Lin H., Chen Q., Yao G., Zhou Y., Zeng F. (2018). Study on luminescent properties of Ce^3+^ sensitized Tb^3+^ doped gadolinium borosilicate scintillating glass. J. Lumin..

[50] Chen Q., Zuo J., He X., Mo X., Tong P., Zhang L. (2017). Enhanced fluorescence of terbium with thiabendazole and application in determining trace amounts of terbium and thiabendazole. Talanta.

[51] Yang Y., Song X., Xu C., Wang Y., Zhang G., Liu W. (2018). A multifunctional and recyclable terbium (iii) coordination polymer: Displaying highly selective and sensitive detection of Fe^3+^ and Cr VI anions, and picric acid in aqueous media. Dalton Trans..

[52] De la Cruz J., Merino R.P., Trejo-García P., Espinosa J., Torres R.A., Moreno-Barbosa E., Gervacio-Arciniega J., Soto E. (2018). Luminescent properties of a hybrid SiO_2_-PMMA matrix doped with terbium. Opt. Mater..

[53] Chen B.B., Liu M.L., Zhan L., Li C.M., Huang C.Z. (2018). Terbium (III) Modified Fluorescent Carbon Dots for Highly Selective and Sensitive Ratiometry of Stringent. Anal. Chem..

[54] Xue S.-F., Chen Z.-H., Han X.-Y., Lin Z.-Y., Wang Q.-X., Zhang M., Shi G. (2018). DNA Encountering Terbium (III): A Smart “Chemical Nose/Tongue” for Large-Scale Time-Gated Luminescent and Lifetime-Based Sensing. Anal. Chem..

[55] Wang Y., Lin S., Luo J., Huang R., Cai H., Yan W., Yang H. (2018). A Novel Tb@ Sr-MOF as Self-Calibrating Luminescent Sensor for Nutritional Antioxidant. Nanomaterials.

[56] Richards B. (2006). Luminescent layers for enhanced silicon solar cell performance: Down-conversion. Sol. Energy Mater. Sol. Cells.

[57] Richards B. (2006). Enhancing the performance of silicon solar cells via the application of passive luminescence conversion layers. Sol. Energy Mater. Sol. Cells.

[58] Enrichi F., Armellini C., Belmokhtar S., Bouajaj A., Chiappini A., Ferrari M., Quandt A., Righini G.C., Vomiero A., Zur L. (2018). Visible to NIR downconversion process in Tb^3+^-Yb^3+^ codoped silica-hafnia glass and glass-ceramic sol-gel waveguides for solar cells. J. Lumin..

[59] Wei Y., He Y., Li X., Chen H., Deng X. (2017). Cellular Uptake and Delivery-Dependent Effects of Tb^3+^-Doped Hydroxyapatite Nanorods. Molecules.

[60] Wang Z., Fang C., Sun Y., YANG H. (2010). Synthesis and characterization of Tb-doped hydroxyapatite fluorescent nanoparticles. Mod. Chem. Ind..

[61] Yin H., Li Y., Bai J., Ma M., Liu J. (2017). Effect of calcinations temperature on the luminescence intensity and fluorescent lifetime of Tb^3+^-doped hydroxyapatite (Tb-HA) nanocrystallines. J. Materiomics.

[62] Qiao Y., LI Y.-X., YIN H.-R., LIU P., LI S.-Y., ZHANG P. (2015). Preparation and Luminescent Properties of Terbium-doped Hydroxyapatite. Chin. J. Lumin..

[63] Yang P., Quan Z., Li C., Kang X., Lian H., Lin J. (2008). Bioactive, luminescent and mesoporous europium-doped hydroxyapatite as a drug carrier. Biomaterials.

[64] Han Y., Wang X., Li S. (2010). Biocompatible Europium Doped Hydroxyapatite Nanoparticles as a Biological Fluorescent Probe. Curr. Nanosci..

[65] Yang C., Yang P., Wang W., Wang J., Zhang M., Lin J. (2008). Solvothermal synthesis and characterization of Ln (Eu^3+^, Tb^3+^) doped hydroxyapatite. J. Colloid Interface Sci..

[66] Doat A., Fanjul M., Pellé F., Hollande E., Lebugle A. (2003). Europium-doped bioapatite: A new photostable biological probe, internalizable by human cells. Biomaterials.

[67] Tesch A., Wenisch C., Herrmann K.-H., Reichenbach J.R., Warncke P., Fischer D., Müller F.A. (2017). Luminomagnetic Eu^3+^- and Dy^3+^-doped hydroxyapatite for multimodal imaging. Mater. Sci. Eng. C.

[68] Mayer I., Layani J.D., Givan A., Gaft M., Blanc P. (1999). La ions in precipitated hydroxyapatites. J. Inorg. Biochem..

[69] Ghosh R., Sarkar R., Paul S. (2016). Development of machinable hydroxyapatite-lanthanum phosphate composite for biomedical applications. Mater. Des..

[70] Wang X., Huang J., Zhang T., Wang K. (2009). Cytoskeleton reorganization and FAK phosphorylation are involved in lanthanum (III)-promoted proliferation and differentiation in rat osteoblasts. Prog. Nat. Sci..

[71] Jadalannagari S., Deshmukh K., Verma A.K., Kowshik R.V., Meenal Ramanan S.R. (2014). Lanthanum-Doped Hydroxyapatite Nanoparticles as Biocompatible Fluorescent Probes for Cellular Internalization and Biolabeling. Sci. Adv. Mater..

[72] Ahymah Joshy M.I., Elayaraja K., Suganthi R.V., Chandra Veerla S., Kalkura S.N. (2011). In vitro sustained release of amoxicillin from lanthanum hydroxyapatite nano rods. Curr. Appl. Phy..

